# Visfatin Enhances Breast Cancer Progression through CXCL1 Induction in Tumor-Associated Macrophages

**DOI:** 10.3390/cancers12123526

**Published:** 2020-11-26

**Authors:** Yen-Yun Wang, Huan-Da Chen, Steven Lo, Yuk-Kwan Chen, Yu-Ci Huang, Stephen Chu-Sung Hu, Ya-Ching Hsieh, Amos C. Hung, Ming-Feng Hou, Shyng-Shiou F. Yuan

**Affiliations:** 1 School of Dentistry, College of Dental Medicine, Kaohsiung Medical University, Kaohsiung 807, Taiwan; wyy@kmu.edu.tw (Y.-Y.W.); yukkwa@kmu.edu.tw (Y.-K.C.); 2 Translational Research Center, Kaohsiung Medical University Hospital, Kaohsiung 807, Taiwan; 1090154@kmuh.org.tw (H.-D.C.); chuntao.hung@uqconnect.edu.au (A.C.H.); 3 Department of Medical Research, Kaohsiung Medical University Hospital, Kaohsiung 807, Taiwan; 4 Center for Cancer Research, Kaohsiung Medical University, Kaohsiung 807, Taiwan; 5 Canniesburn Regional Plastic Surgery and Burns Unit, Glasgow Royal Infirmary, Glasgow G4 0SF, UK; Steven.Lo@ggc.scot.nhs.uk; 6 College of Medical, Veterinary and Life Sciences, University of Glasgow, Glasgow G12 8QQ, UK; 7 Division of Oral Pathology & Maxillofacial Radiology, Kaohsiung Medical University Hospital, Kaohsiung 807, Taiwan; 8 Oral & Maxillofacial Imaging Center, College of Dental Medicine, Kaohsiung Medical University, Kaohsiung 807, Taiwan; 9 Graduate Institute of Medicine, College of Medicine, Kaohsiung Medical University, Kaohsiung 807, Taiwan; u109500044@gap.kmu.edu.tw; 10 Department of Dermatology, College of Medicine, Kaohsiung Medical University, Kaohsiung 807, Taiwan; 940064@kmuh.org.tw; 11 Department of Dermatology, Kaohsiung Medical University Hospital, Kaohsiung 807, Taiwan; 12 Department of Dermatology, Kaohsiung Municipal Siaogang Hospital, Kaohsiung 812, Taiwan; 13 Institute of Cancer Sciences, University of Glasgow, Glasgow G61 1BD, UK; ya-ching.hsieh@glasgow.ac.uk; 14 Division of General and Gastroenterological Surgery, Department of Surgery, Kaohsiung Medical University Hospital, Kaohsiung 807, Taiwan; mifeho@kmu.edu.tw; 15 Department of Biological Science and Technology, College of Biological Science and Technology, National Chiao Tung University, Hsinchu 300, Taiwan; 16 Center for Intelligent Drug Systems and Smart Bio-Devices (IDS2B), National Chiao Tung University, Hsinchu 300, Taiwan

**Keywords:** visfatin, breast cancer, tumor-associated macrophages, CXCL1

## Abstract

**Simple Summary:**

Visfatin is an adipocytokine highly expressed in breast tumor tissues and circulation, and is positively associated with breast cancer progression and poorer clinical prognosis. In this study, we explored the role of adipocytokine in the breast tumor microenvironment with the focus on the interactions between visfatin and macrophages in breast cancer development by using in vitro, in vivo, and clinical studies. Visfatin promoted M2 differentiation in monocytic cells through ERK/CXCL1 induction and enhanced breast cancer cell viability, migration, tumorsphere formation, EMT, and stemness. Our study adds new insights to the growing body of evidence supporting the role of tumor-stromal interactions in breast cancer and also provides a potential therapeutic target.

**Abstract:**

Visfatin, an adipocytokine highly expressed in breast tumor tissues, is associated with breast cancer progression. Recent studies showed that adipocytokines mediate tumor development through adipocytokine tumor-stromal interactions in the tumor microenvironment. This study focused on the interaction between one key stromal constituent—tumor-associated macrophages—and visfatin. Pretreatment of THP-1 and peripheral blood mononuclear cells (PBMCs) with recombinant visfatin resulted in M2-polarization determined by CD163 and CD206 expression. Indirect co-culture with visfatin-treated THP-1 (V-THP-1) promoted the viability, migration, tumorsphere formation, EMT, and stemness of breast cancer cells. Cytokine array identified an increased CXCL1 secretion in V-THP-1 conditioned medium and recombinant CXCL1 enhanced cell migration and invasion, which were abrogated by the CXCL1-neutralizing antibody. Additionally, visfatin induced pERK in THP-1 cells and clinical samples confirmed a positive CXCL1/pERK correlation. In an orthotopic mouse model, the tumor bioluminescent signal of luciferase-expressing MDA-MB-231 (Luc-MDA-MB-231) cells co-cultured with V-THP-1 and the expression of proliferation marker Ki67 were significantly higher than that co-cultured with THP-1. Furthermore, tail vein-injected Luc-MDA-MB-231 pretreated with V-PBMCs conditioned medium metastasized to lungs more frequently compared to control, and this was reversed by CXCL1 blocking antibody. In summary, this study demonstrated that visfatin enhanced breast cancer progression via pERK/CXCL1 induction in macrophages.

## 1. Introduction

Breast cancer, one of the most frequent malignant diseases worldwide, has several well-explored risk factors, including age, gene mutations, metabolic disorder, and exogenous hormone usage [1]. Obesity-related cytokines, also known as adipocytokines, are lesser-known but intriguing potential risk markers in breast cancer. Adipocytokines are associated with poorer clinical prognosis and inferior treatment response, although the exact pathobiological mechanisms remain unclear [2,3,4,5,6].

Adipocytokines may affect breast tumor cell proliferation, invasion, and migration through autocrine, paracrine, and endocrine pathways [7]. Of particular interest is the presence and interplay of adipocytokines in the breast tumor microenvironment, with high levels of leptin, resistin, and visfatin noted in Taiwanese breast cancer patients [8,9,10]. The breast tumor microenvironment is known to play a vital role in breast cancer progression and metastatic behavior [11], with tumor cells interacting with several adjacent cell types, including adipocytes, immune cells, mesenchymal stem cells, fibroblasts, myofibroblasts, blood vessels, and the extracellular matrix. This microenvironment interplay is effected through paracrine signaling pathways that may influence epithelial-mesenchymal transition (EMT) and stemness in cancer cells, directly impacting upon tumor progression and metastasis [12].

Immune cells are key players in the tumor microenvironment, with macrophages differentiating into two types of tumor-associated macrophages (TAMs): M1-like and M2-like TAMs. They differ in their response toward tumor cells, with M1-like TAMs promoting immunosurveillance to eradicate cancer cells, while M2-like TAMs suppress immunosurveillance [13,14,15,16]. By producing immunosuppressive factors such as TGF-β and IL-10, and recruitment of immunosuppressive T regulatory cells via chemokine CCL22 production, M2-like TAMs suppress the immune surveillance toward cancer cells [17,18,19]. Furthermore, M2-like TAMs create a tumor-promoting microenvironment through matrix metalloprotease remodeling of the extracellular matrix, thus promoting tumor angiogenesis, invasion, and metastasis [13,20,21]. In addition to generating immunosuppressive cytokines, TAMs produce proinflammatory cytokines including TNFα, IL-1, and IL-6 that initiate NF-κB signal transduction, which is a critical factor in cancer cell inflammation. Other than its role in the inflammatory response, NF-κB participates in cancer cell stemness maintenance, tumor development, and resistance to tumor therapy [22,23,24,25,26,27].

Visfatin, also known as nicotinamide phosphoribosyl transferase (NAMPT) or pre-B-cell colony-enhancing factor (PBEF) [28], exhibits differing intra/extracellular roles. Intracellular visfatin acts as a rate-limiting enzyme in nicotinamide adenine dinucleotide (NAD) biosynthesis, while extracellular visfatin is involved in NAD formation or acts as a cytokine [29]. In tumor development, visfatin may enhance metastasis and angiogenesis through PI3K/Akt, MAPK, and c-Abl/STAT3 signaling pathways in macrophages, endothelial cells, and breast cancer cells. Furthermore, visfatin has been reported to promote breast cancer cell growth via the NF-κB/Notch1 pathway [30,31,32].

According to our previous studies, high levels of visfatin in breast cancer tissue act as a marker for tumor progression and poor prognosis [9]. More recently, we have demonstrated that visfatin not only directly influences breast cancer progression but also indirectly affects breast cancer through GDF-15 secretion in ADSCs, thus promoting breast cancer malignant behavior [33]. Additionally, visfatin induces the production of inflammatory cytokines, upregulation of co-stimulatory molecules, and augmentation of phagocytosis in CD14^+^ monocytes [34]. Currently, there are no data regarding the role of visfatin in TAMs within the breast tumor microenvironment. However, in chronic lymphocytic leukemia (CLL), visfatin promoted CLL monocyte differentiation towards M2 phenotype [35,36], and is involved in human monocytic cell THP-1 differentiation via PMA pretreatment [37]. Therefore, the present study examined the interplay between visfatin and the breast tumor microenvironment, focusing on the role of TAMs as an intermediary in breast cancer progression.

## 2. Results

### 2.1. Visfatin Secreted by Breast Cancer Cells Induced Macrophage Differentiation in THP-1 and PBMCs

**Figure 1:**
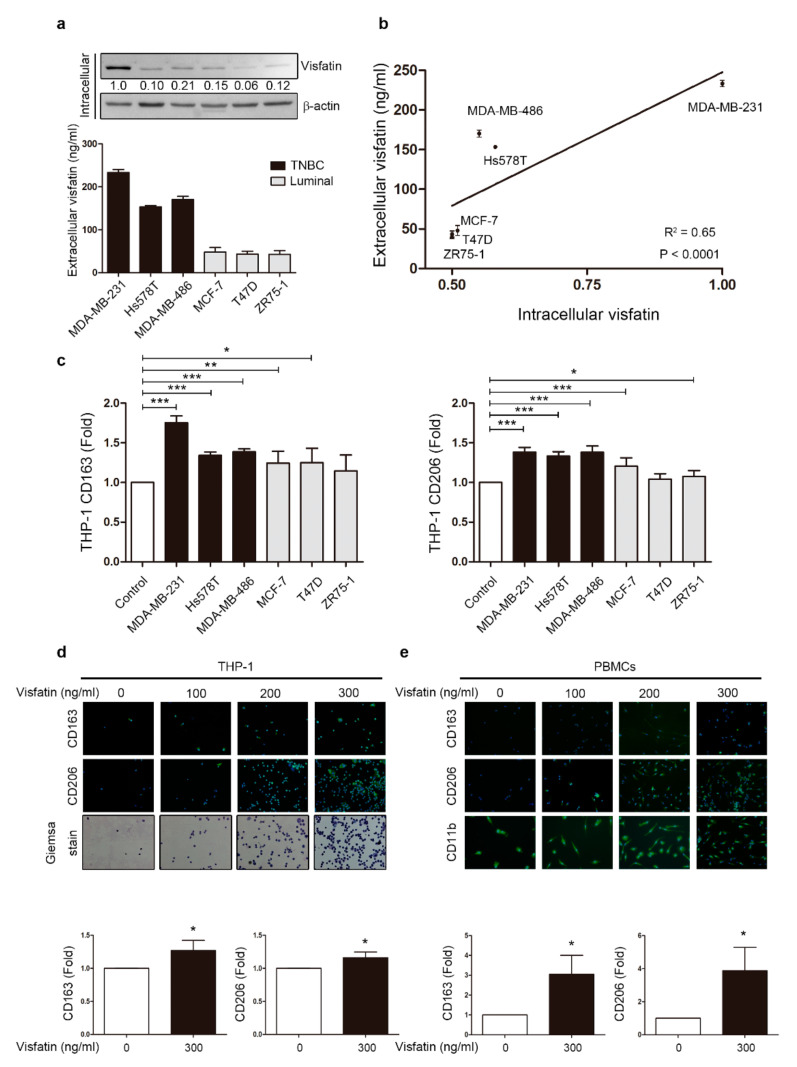
Breast cancer cell-derived visfatin induced THP-1 and PBMCs differentiation. (**a**) Breast cancer cells were harvested after culture for 48 h, and intracellular visfatin expression was analyzed by Western blot and extracellular visfatin level by ELISA. Breast cancer cells included triple-negative breast cancer (TNBC) cells (MDA-MB-231, Hs578T, and MDA-MB-486) and luminal breast cancer cells (MCF-7, T47D, and ZR75-1). (**b**) Correlation between intra/extracellular visfatin was analyzed, with each point representing one breast cancer cell line. (**c**) Conditioned medium (CM) refers to the medium collected after breast cancer cell culture. Human monocytic cell line THP-1 was treated with CM from various breast cancer cell lines for six days, and M2 markers CD163 and CD206 were analyzed by flow cytometry. (**d**) THP-1 cells were treated with visfatin at different doses (0, 100, 200, 300 ng/mL) for six days, and M2 markers CD163 and CD206 were detected in attached cells by immunofluorescence staining. Giemsa stain was used to identify macrophages. (40×) (**e**) PBMCs were treated with visfatin at different doses (0, 100, 200, 300 ng/mL) for six days, and M2 markers CD163 and CD206 were detected in attached cells by immunofluorescence staining, with CD11b as a macrophage marker. Statistical analysis was performed using the *t*-test and the correlation analysis by linear regression (40×). *p*-value ≤ 0.05 marked as *; *p*-value ≤ 0.01 marked as **; *p*-value ≤ 0.001 marked as ***.

We focused our analysis on visfatin, an oncoprotein highly expressed in the serum of breast cancer patients [33]. First, endogenous visfatin expression at different stages of breast cancer cells was examined by Western blot and ELISA. Intracellular visfatin protein levels appeared to positively correlate with the malignancy of the breast cancer cells by Western blot, as did extracellular visfatin levels in the conditioned medium (CM) analyzed by ELISA (Figure 1a and Figure S6). The analysis also showed a statistically positive correlation between intracellular and extracellular visfatin expressed by breast cancer cells (Figure 1b). In previous studies, visfatin has been shown to promote breast cancer malignant behaviors such as migration, invasion, and metastasis [33]. However, it is unclear whether visfatin secreted from breast cancer cells can directly affect the differentiation stage of immune cells recruited to the tumor microenvironment. Macrophages have high plasticity, enabling phenotype shift in response to numerous environmental stimuli [38]. Tumor environmental factors influence the differentiation of TAMs into the M2 phenotype. In this regard, as visfatin is highly expressed in the breast tumor microenvironment, the question remains whether visfatin may affect macrophage phenotype [9]. Therefore, CM collected from different types of breast cancer cells were used to treat human monocytic cell THP-1, and M2-like macrophage markers were observed by flow cytometry (Figure 1c and Figure S1a). Notably, two representative M2 markers, CD163 and CD206, were increased after treatment with the CM from breast cancer cells, especially the triple-negative breast cancer cell MDA-MB-231. Moreover, using an indirect trans-well system in which THP-1 was co-cultured with various breast cancer cell lines that mimicked the tumor microenvironment, we observed an increase in CD163 in the MDA-MB-231 group (Figure S1b). These results suggest that breast cancer cells can indirectly influence THP-1 to differentiate into M2-like macrophages by the secretome, including visfatin.

Next, to evaluate the direct effect of visfatin on THP-1, THP-1 cells and PBMCs were treated with recombinant protein visfatin. After six days of treatment, THP-1 cells showed a more M2-like differentiation with increasing dosage of visfatin by observing CD163 and CD206 expression with immunofluorescent staining (Figure 1d), and the same pattern was observed in PBMCs treated with visfatin (Figure 1e). According to previous studies, visfatin induced THP-1 cells to differentiate into M2 macrophages only if THP-1 were under differentiating stage triggered by PMA [37]. Here we found that visfatin induced THP-1 differentiation into M2 macrophages over a long-term period with or without PMA treatment, since the expression levels of CD163 and CD206 in both groups were significantly increased (Figure 1d,e and Figure S2). These results raise the possibility that breast cancer cells may modulate visfatin expression to enhance malignancy through monocyte differentiation.

### 2.2. Visfatin-Treated THP-1 Cells Promoted Malignant Behaviors in Breast Cancer Cells

In our previous report, expression of visfatin was elevated in breast cancer tissues compared to adjacent healthy breast tissues by immunohistochemistry (IHC) [9]. To explore whether visfatin promoted progression of different subsets of breast cancer cells through stimulation of tumor stromal cells such as macrophages, we indirectly co-cultured human monocytic cell line THP-1 with breast cancer cell lines MDA-MB-231 and MCF-7 for 24 h after pretreatment with or without visfatin 300 ng/mL for six days. Visfatin concentration was based on previous studies [4,28,37]. MDA-MB-231 and MCF-7 breast cancer cells were not the same in terms of breast cancer cell characteristics because of the different HER2, estrogen receptor (ER), and progesterone receptor (PR) expression patterns in these cells. These three markers were associated with the malignant behavior of breast cancer cells. Generally, loss of HER2, ER, and PR expression refers to as triple-negative breast cancer cells (e.g., MDA-MB-231), while ER and PR positive cells were less malignant as luminal A breast cancer cells (e.g., MCF-7). TNBCs and luminal A breast cancer cells showed different phenotypes in both clinical prognosis and tumor aggressiveness [39]. After co-culture, breast cancer cell lines were collected for further malignant behavior analysis, including cell viability, invasion, migration, and tumorsphere formation. Higher cell viability was found in MDA-MB-231 and MCF-7 cell lines in the V-THP-1 co-culture group compared to THP-1 alone on days 1 and 2 (Figure S3a); however, only MDA-MB-231 showed higher invasion ability while MCF-7 showed no significant difference with or without visfatin treatment (Figure S3b).

**Figure 2:**
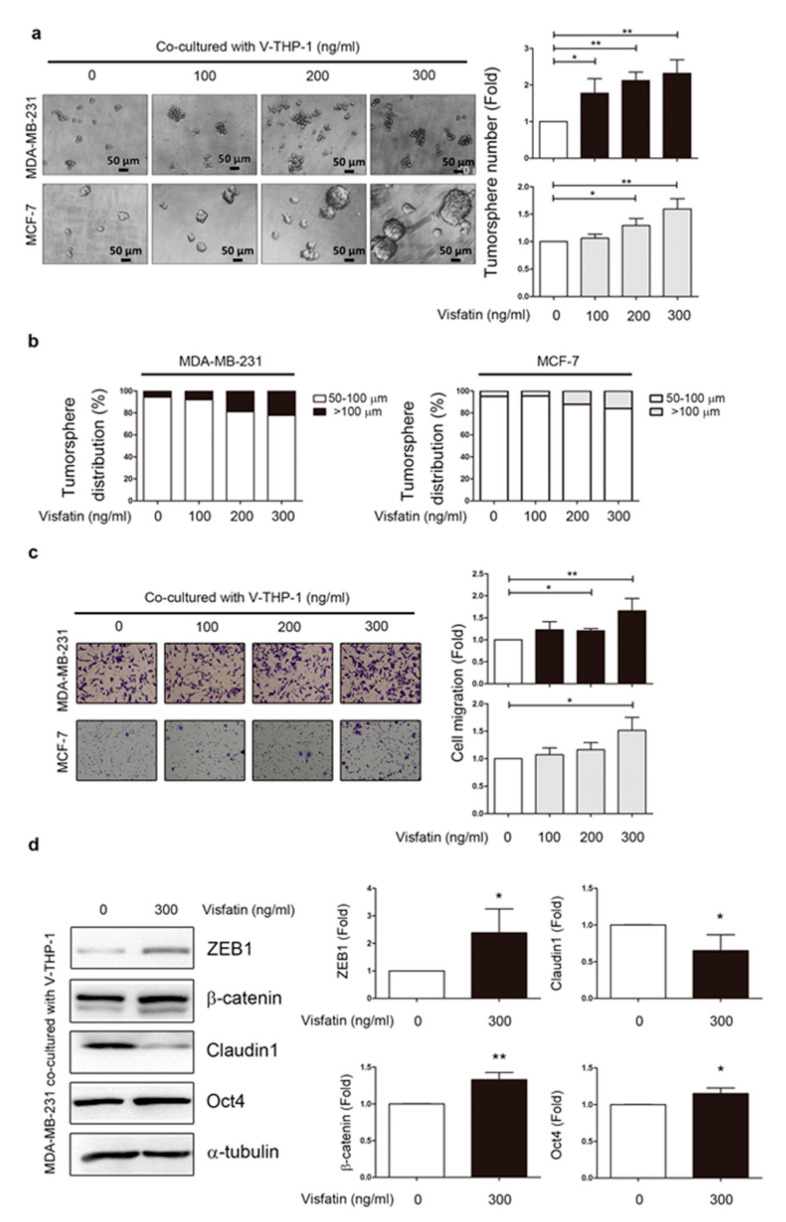
Visfatin-treated THP-1 enhanced tumor development in breast cancer cells. (**a**) MDA-MB-231 and MCF-7 were co-cultured with V-THP-1 at different doses (0, 100, 200, 300 ng/mL) for two days, and tumorsphere formation was observed by monitoring the size of the suspension cell clusters for another seven days. Cell clusters with size over 50 μm were counted as positive tumorsphere formation. Image scale bar, 50 μm. (**b**) Tumorsphere sizes defined as 50–100 μm and ≥100 μm were plotted in a bar chart. (**c**) MDA-MB-231 and MCF-7 cells were co-cultured with visfatin-treated THP-1 at different doses (0, 100, 200, 300 ng/mL) for 24 h, and migration ability was assessed in a trans-well system (40×). (**d**) MDA-MB-231 was co-cultured with visfatin-treated THP-1 (0, 300 ng/mL) for 24 h, and EMT and stemness markers were evaluated by Western blotting. Statistical analysis was done by the *t*-test. *p*-value ≤ 0.05 marked as *; *p*-value ≤ 0.01 marked as **.

Both breast cancer cell lines showed significantly increased number of spheroid formation (≥50 μm) after co-culture with V-THP-1 compared to THP-1 alone group (Figure 2a). As the overall number of ≥50 μm tumorspheres increased, the population of >100 μm tumorspheres also gradually increased (Figure 2b). Furthermore, the migration ability of MCF-7 and MDA-MB-231 was significantly enhanced after co-culture with V-THP-1 compared to THP-1 alone group (Figure 2c). Since migration increased under visfatin-treatment conditions, we also evaluated EMT and stemness markers by Western blot in breast cancer cells co-cultured with or without visfatin-treated THP-1 cells. Mesenchymal markers ZEB-1 and β-catenin and stemness marker OCT-4 were increased while epithelial marker claudin was decreased (Figure 2d and Figure S7). In summary, these data indicate that indirect co-culture of V-THP-1 with breast cancer cells enhanced malignant behaviors, including viability, migration, and tumorsphere formation.

### 2.3. Visfatin Induced CXCL1 Secretion from THP-1 Cells Which Promoted Breast Cancer Cell Metastasis

**Figure 3:**
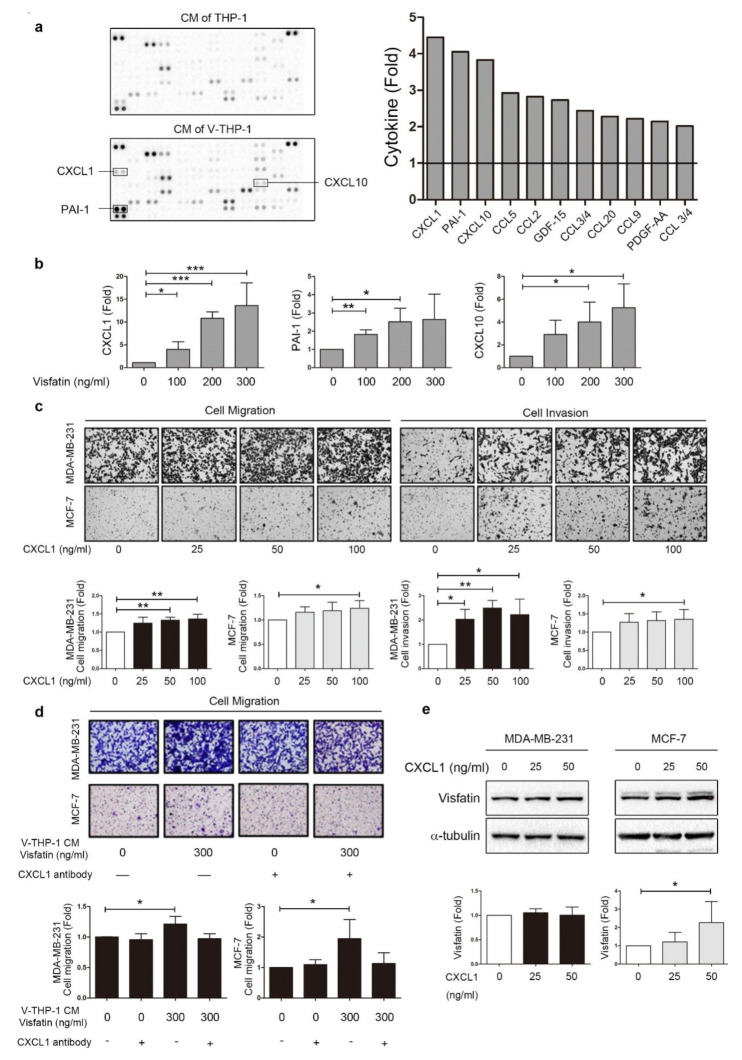
Visfatin induced CXCL1 secretion from THP-1 cells which promoted breast cancer cell malignant behavior. (**a**) THP-1 cells were treated with visfatin (0, 300 ng/mL) for 24 h, and CM was collected for the cytokine-chemokine array. The top 10 cytokines were listed in the bar chart. (**b**) ELISA confirmed the top three significantly increased cytokines. THP-1 cells were treated with visfatin at different doses (0, 100, 200, 300 ng/mL), and the conditioned medium was collected to detect CXCL1, PAI-1, and CXCL10. (**c**) MDA-MB-231 and MCF-7 were treated with recombinant protein CXCL1 at different doses (0, 25, 50, 100 ng/mL) in the trans-well system, and migration and invasion assays were performed (40×). (**d**) MDA-MB-231 and MCF-7 were treated with conditioned medium from V-THP-1 (0, 300 ng/mL) combined with or without CXCL1 blocking antibody, and migration assay was performed. The quantitative analysis was shown in the bar chart (40×). (**e**) MDA-MB-231 and MCF-7 were treated with recombinant protein CXCL1 at different doses (0, 25, 50 ng/mL) for three days, and visfatin expression level was detected by Western blot. The *t*-test was used for statistical analysis. *p*-value ≤ 0.05 marked as *; *p*-value ≤ 0.01 marked as **; *p*-value ≤ 0.001 marked as ***.

Multiple components in the supernatant may influence the activity of breast cancer cells, such as cytokines/chemokines, growth factors, and metabolites. Further analysis was performed on the expression pattern of cytokines/chemokines in the secretion by using an antibody array kit (Figure 3a). After analysis, several cytokines/chemokines were found to be elevated in the supernatant of visfatin-treated THP-1 compared to untreated cells, with chemokine (C-X-C motif) ligand 1 (CXCL1), plasminogen activator inhibitor type-1 (PAI-1), and chemokine (C-X-C motif) ligand 10 (CXCL10) as the top three elevated candidates. CXCL1 has been found to positively correlate with colon cancer progression [40], and poor prognosis in breast cancer [41]; PAI-1 also plays an important role in tumor progression, invasion, and metastasis in breast cancer [42,43]. On the other hand, CXCL10 plays an antitumor role compared to CXCL1 and PAI-1 [44,45]. Next, we reconfirmed the results from the cytokines-chemokines array by ELISA. CXCL1 was dramatically increased approximately 13-fold following visfatin 300 ng/mL treatment, compared to PAI-1 and CXCL10 at approximately threefold to fivefold (Figure 3b). Although secretion of anti-tumor cytokines CXCL10 and CCL5 (ranking third and fourth in cytokine array) from THP-1 was increased by visfatin treatment, other tumor progression factors triggered by V-THP-1, especially the two top-ranking cytokines CXCL1 and PAI-1 in cytokine array, had a protumor property and led to breast tumor progression eventually.

The ELISA results indicated that the sensitivity of CXCL1 toward visfatin was higher than PAI-1 (Figure 3b). Therefore, we focused on the role of CXCL1 in breast cancer cells. MCF-7 and MDA-MB-231 cell lines were treated with recombinant protein CXCL1 and performed by migration and invasion assays. CXCL1 treatment increased invasion ability of MCF-7 and MDA-MB-231 in a dose-dependent manner; however, the migration ability of MCF-7 and MDA-MB-231 was enhanced only at high concentrations of CXCL1 (Figure 3c). The enhancement of malignant behavior by V-THP-1 CM was significantly reversed by treatment with CXCL1 blocking antibody (Figure 3d). CXCL1 may induce visfatin production to form a positive feedback loop, which is supported by the finding that treatment of breast cancer cells with CXCL1 recombinant protein resulted in significantly increased visfatin expression in MCF-7, but not MDA-MB-231 cells (Figure 3e and Figure S8). These results indicate that CXCL1 produced by V-THP-1 is involved in breast cancer progression, and may mediate the cross-talk between macrophages and breast cancer cells in a positive feedback regulation.

### 2.4. ERK Phosphorylation Is Required for CXCL1 Secretion from Visfatin-Treated THP-1 Cells

**Figure 4:**
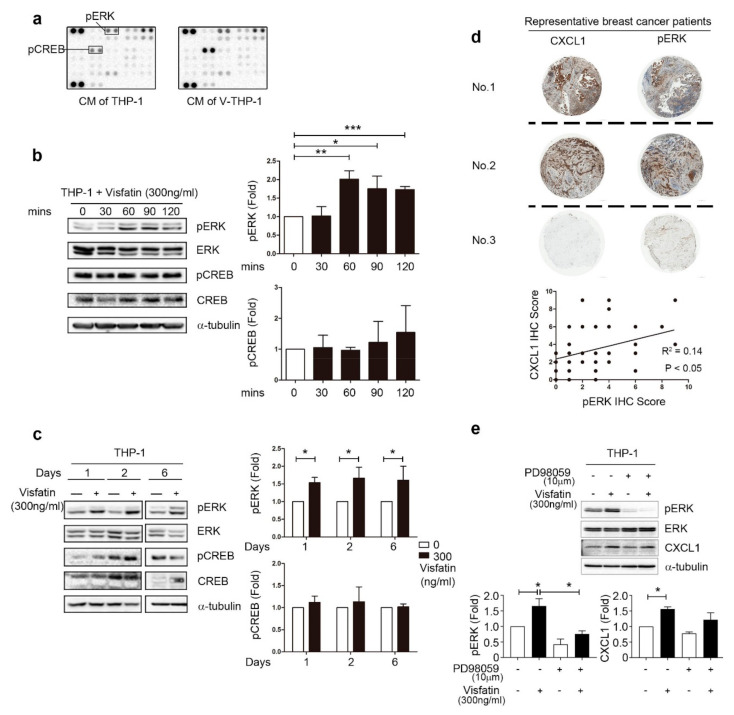
Visfatin induced ERK phosphorylation in THP-1 cells, which positively correlated with CXCL1 in clinical patients. (**a**) THP-1 cells were treated with visfatin (0, 300 ng/mL) for one hour, and CM was collected for phospho-kinase array. (**b**) THP-1 cells were treated with visfatin (0, 300 ng/mL) for short-term period (0, 30, 60, 90, 120 min), and phosphorylation of ERK and CREB were detected by Western blot. (**c**) THP-1 cells were treated with visfatin (0, 300 ng/mL) for a long-term period (one, two, six days), and phosphorylation of ERK and CREB was detected by Western blot. (**d**) Human breast tumor tissue microarray from clinical breast cancer patients was analyzed by IHC staining with CXCL1 and phospho-ERK (40×). (**e**) THP-1 cells were pretreated with visfatin (300 ng/mL) for six days, and PD98059 (10 μM) was added on day 3. CXCL1 protein level was increased while visfatin treatment, but was decreased by ERK phosphorylation inhibitor. Statistical analysis was performed by the *t*-test and the correlation analysis by linear regression. *p*-value ≤ 0.05 marked as *; *p*-value ≤ 0.01 marked as **; *p*-value ≤ 0.001 marked as ***.

The CXCL signaling pathway was further explored using phospho-kinase array kit. The expression levels of pERK and pCREB were upregulated in THP-1 after treatment with visfatin for one hour (Figure 4a). We also confirmed the expression level of pERK, but not pCREB, was upregulated in V-THP-1, and the high level of pERK could be maintained short-term (Figure 4b and Figure S9) and up to six days compared to untreated control cells (Figure 4c and Figure S10). Lower concentration of visfatin that better mimics the human in vivo situation was further evaluated, and ERK phosphorylation continued to be present when compared to the visfatin-free group (Figure S4). In previous studies, CXCL1 has been reported to correlate with ERK phosphorylation in breast cancer, colorectal cancer, and LPS-induced inflammation [46,47,48]. To broadly investigate the correlation between CXCL1 and pERK in clinical patients, we analyzed tissue microarray from clinical patients. To more precisely examine the effect of macrophages on breast tumors, we selected immune cells within breast cancer tissue for CXCL1 and pERK IHC signal analysis. There was a statistically positive correlation between CXCL1 and pERK expression within the immune cells inside the breast tumor (Figure 4d). Moreover, in V-THP-1, pERK inhibitor (PD98059) suppressed the expression levels of pERK as well as CXCL1 (Figure 4e and Figure S11). In summary, CXCL1 expression and secretion induced in V-THP-1 contributed to the malignancy of breast cancer cells and positively correlated with ERK phosphorylation in the visfatin-TAMs-CXCL1 axis.

### 2.5. Visfatin-Treated THP-1 Enhanced MDA-MB-231 Tumor Formation and Metastasis in the Orthotopic and Tail Vein-Injected Xenograft Mouse Models

**Figure 5:**
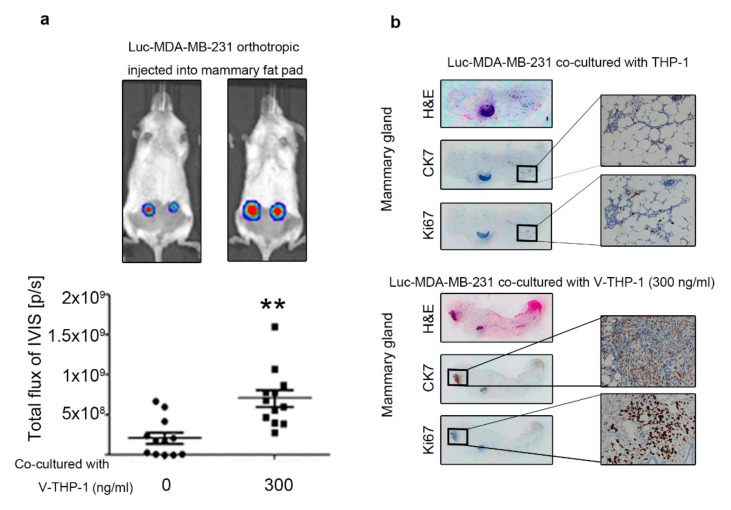
MDA-MB-231 co-cultured with visfatin-treated THP-1 showed enhanced tumor formation in vivo. (**a**) Luc-MDA-MB-231 breast cancer cells co-cultured with visfatin-treated (0, 300 ng/mL) THP-1 cells were injected orthotopically in the fourth mammary gland of NOD/SCID mice, and luminescence signal was observed by IVIS system. (**b**) Mammary gland tissue was analyzed by H&E staining and IHC staining, with CK7 as an epithelial marker for exogenous breast cancer cells, and Ki67 as a cell proliferation marker (40× (**left**) and 200× (**right**)). The *t*-test was used for statistical analysis. *p*-value ≤ 0.01 marked as **.

To confirm the in vitro findings, we further examined in vivo mouse model equivalents. Firstly, Luc-MDA-MB-231 were collected after co-culture with or without V-THP-1 and injected into the mouse mammary gland, and subsequent tumor development was assessed through the IVIS system. The IVIS signal in the mammary gland was significantly increased in the V-THP-1 group compared to THP-1 alone (Figure 5a and Figure S5a). By using IHC staining, cell proliferation marker Ki67 expression was significantly increased in the V-THP-1 group compared to THP-1 group, as well as the H&E staining signal (Figure 5b). To confirm that tumor mass was derived from exogenous breast cancer cells that we injected, we chose epithelial marker CK7, which has been reported as a triple-negative breast cancer cell marker [49,50,51].

To rapidly examine the effect of CM of V-PBMCs on MDA-MB-231 metastatic ability and for the challenges of maintaining a specific visfatin-enriched tumor microenvironment for a longer period of time in mice, we therefore decided to carry out the tail-vein metastasis model rather than the spontaneous metastasis model [52,53].

**Figure 6:**
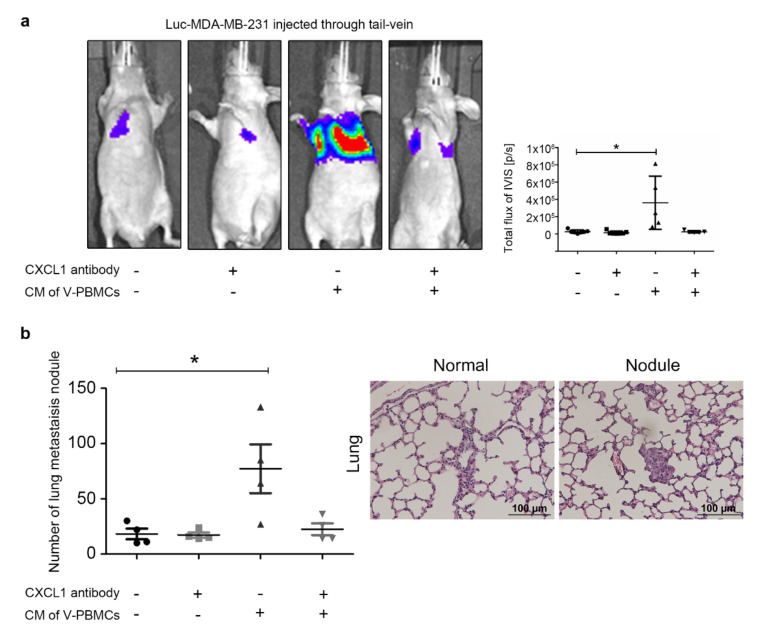
MDA-MB-231 pretreated with CM from V-PBMCs showed enhanced cancer cell metastasis, which was inhibited by CXCL1 antibody. (**a**) Luc-MDA-MB-231 breast cancer cells were treated with or without CM from V-PBMCs (300 ng/mL) and then tail-vein injected into nude mice. The CXCL1 blocking antibody (10 mg/kg) was intraperitoneally injected into mice twice weekly after a week later, and luminescence signal was observed by IVIS system. (**b**) After sacrifice, we harvested the lung and analyzed the metastatic tumor nodules in the lung tissue by H&E staining. A representative image of normal tissue and nodule was presented. The *t*-test was used for statistical analysis. *p*-value ≤ 0.05 marked as *.

In Figure 6, Luc-MDA-MB-231 cells were pretreated with or without CM from V-PBMCs, followed by injection into the tail-veins of NOD/SCID mice. The IVIS signal was significantly increased in the lung of the V-PBMCs pretreatment group, and this was reversed by CXCL1 blocking antibody treatment (Figure 6a and Figure S5b). After sacrifice, lung tissue was collected for metastatic nodule analysis using H&E staining. Similar to the results of the IVIS bioluminescence signal, significantly increased metastatic nodules were identified in Luc-MDA-MB-231 pretreated with CM of V-PBMCs. However, the number of nodules was decreased in the presence of CXCL1 blocking antibody. Of note, the size of the nodules in these groups usually reached about 100 μm, which was categorized as micro nodule type (Figure 6b). Taken together, CXCL1 plays a vital role in breast cancer cell progression and metastasis under the influence of visfatin-treated tumor-associated macrophages.

The mouse model we used in the current study was immunodeficient as the xenografted breast cancer cells were different species from the NOD/SCID mouse host, leading to the difficulty of examining innate immune actions. We noticed that the responses to visfatin treatment in murine mammary cancer cells have not been well determined, neither the responses to co-culture with visfatin-treated murine monocytic cells. Therefore, we did not carry out in vivo experiments by xenografting murine mammary cancer cells in immunocompetent mice in this study. Nevertheless, for enduring objects in visfatin research, it should be adequately addressed in the future studies in order to better understand the roles of PBMCs in this context.

## 3. Discussion

The data presented here indicate for the first time—using a unique visfatin-human monocyte co-culture model—that visfatin may indirectly influence breast tumor progression, invasion, and metastasis, through a THP-1 specific ERK-CXCL1 pathway. This adds to the growing body of evidence supporting the role of tumor-stromal interactions in breast cancer, in particular our previous work on the role of visfatin and adipose-derived stem cells, and the role of obesity and adipocytokines in breast cancer progression [33]. The cross-talk between tumor microenvironment and immune alterations (e.g., T cell-mediated anti-tumor response) indeed influenced tumor progression through many other factors such as IL-1α, IL-10, PD-L1, TNF-α, TGF-β, and CXCL1, 5, 8, 12 [54,55,56], but there was less information about the adipocytokines in this field with consideration of the tumor-immune microenvironment. Here we demonstrate that visfatin can act via biologically distinct pathways from those previously discovered using tumor cell line models in isolation [36].

**Figure 7:**
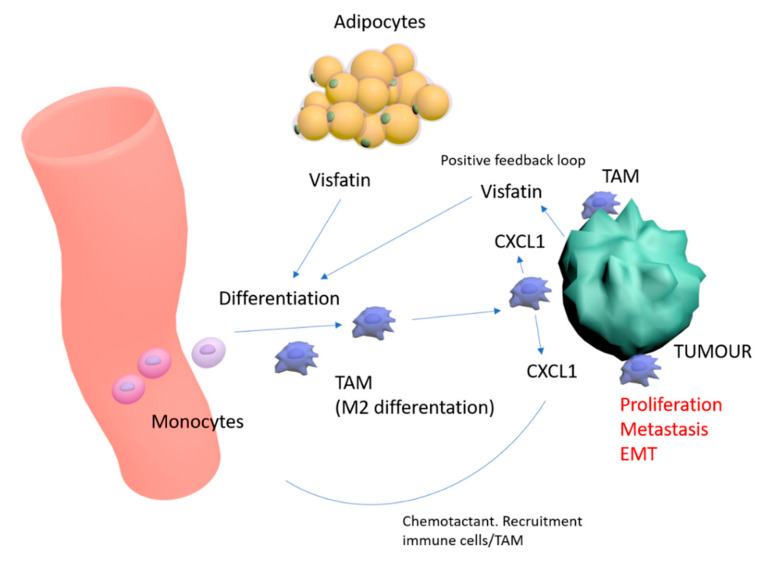
Schematic representation of visfatin effects in the breast tumor microenvironment—and differentiation of monocytes towards M2/TAM phenotype. CXCL1 increases malignant characteristics of breast tumor, and provides a positive feedback loop for increasing visfatin and recruitment of immune cells/TAMs.

In the present study, we noted that visfatin induced human monocyte cell line THP-1 differentiation into M2 macrophages—known to suppress immuno-surveillance and potentially to enhance malignant behaviors. Further co-culture of visfatin-pretreated THP-1 cells with breast cancer cell lines confirmed these effects, with increased tumor viability, migration, and tumorsphere formation when compared to co-culture with untreated THP-1 cells. Further analysis of the supernatant of V-THP-1 noted an increase in several cytokine-chemokines, with CXCL1 exhibiting the greatest comparative increase. We noted that recombinant CXCL1 increased invasion and migration in breast cancer cell lines, with CXCL1 blocking antibody abolishing the enhancement of malignant behavior by V-THP-1 CM. This pathway may be dependent on ERK phosphorylation, with our data indicating the abrogation of CXCL1 protein increases in V-THP-1 cells with pERK inhibition. Recent research indicates that CXCL1 from tumor-associated macrophages acts via downstream NFKB/SOX4 signaling in breast cancer [57,58]. Together, these results indicate that the cross-talk between macrophages and breast cancer cells in the premetastatic niche may be indirectly mediated by CXCL1 triggered by V-THP-1, resulting in breast cancer progression (Figure 7).

The chemokine (C-X-C motif) ligand 1 (CXCL1), previously referred to as the GRO1 oncogene, belongs to a chemokine family that is highly expressed and secreted during inflammation and involved in tumor angiogenesis [59,60,61]. CXCL1 may act as a prognostic marker for several cancers including breast cancer [48,62,63,64], and has been correlated with tumor cell survival and metastasis [65,66,67,68]. CXCL1 has been found to be highly expressed within breast cancer stroma and is inversely associated with TGF-β signal protein, with TGF-β shown to negatively regulate CXCL1 expression [41,69,70]. In ER-negative breast cancer, CXCL1 enhances cancer cell migration and invasion through an ERK/MMP2/MMP9 signal pathway [48]. Likewise, our findings from cytokine-chemokine and phospho-kinase arrays showed positive correlation between CXCL1 and ERK phosphorylation. While others have shown that CXCL1 is highly expressed within breast tumor and stromal cells, including immune cells and carcinoma-associated fibroblasts (CAF), the critical upstream pathways have not been clearly delineated in breast cancer [57,58,70]. Studies in disparate fields, including diabetes and UV irradiation of melanocytes, point towards several upstream signaling proteins, such as NF-κB, STAT1, and TLR-4 [71,72]. Significantly, the present study highlights one of the first potential upstream candidates in breast cancer in the form of the visfatin-monocyte-CXCL1 pathway.

Our study supports existing data that tumor-associated macrophages (TAMs) play a vital role in CXCL1-induced cancer progression [57,73]. Notably, CXCL1 has been shown to recruit tumor-adjacent immune cells to support tumor development, providing a mechanistic form of positive feedback, and can likewise recruit other stromal cells to create a premetastatic niche that supports cancer growth and metastasis [58]. More specifically, we noted that CXCL1 might elevate visfatin levels via a positive feedback loop, with recombinant CXCL1 treatment of breast cancer cells resulting in significantly increased visfatin expression. Together, this highlights a number of critical potential therapeutic targets. Firstly, targeting of downstream CXCL1 may facilitate the disruption of both immune cell recruitment and the visfatin positive feedback loop. Secondly, targeting of upstream visfatin may reduce monocyte polarization to M2 phenotype and concomitant reduction in CXCL1 expression. CXCR2, the receptor for CXCL1, provides one such therapeutic strategy with CXCR2 inhibitors such as Repertaxin currently in Phase II clinical trials in breast cancer [74]. Visfatin, quantifiable in patient’s serum, thus represents both a potential clinical marker for both therapeutic stratification and treatment response.

**Figure 8:**
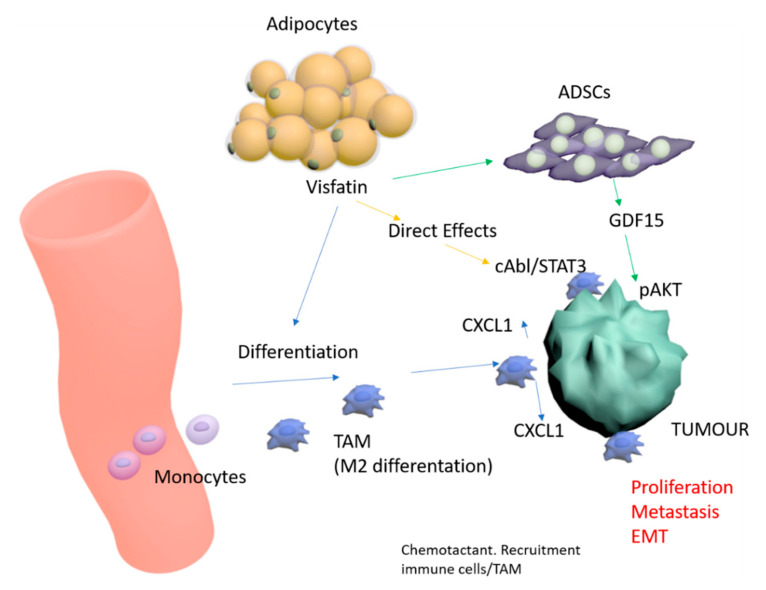
Proposed model of visfatin direct and indirect effects in the context of the breast tumor microenvironment.

A clearer picture is emerging of the complex effects of adipocytokines in breast cancer. Our unique model of visfatin-pretreated monocyte cells co-culture with breast cancer cell lines presented here builds on our prior work with visfatin-pretreated ADSCs co-culture, and strengthens insights into novel tumor-stromal interactions. We have previously demonstrated direct tumor-promoting effects of visfatin on breast tumor cells mediated via c-Abl and STAT3 [36], and indirect effects via ADSC intermediaries acting via a GDF15-pAKT pathway [33]. We outline a proposed model for visfatin in the breast tumor microenvironment that collates these multiple pathways (Figure 8). However, some areas still remain unclear, in particular the role of visfatin in inflammation and cancer stemness. Visfatin has been shown to increase cancer cell proliferation through activation of PI3K/AKT, ERK, p38, and NF-κB signaling pathways. Binding sites for several transcription factors, including NF-κB, AP-1, and NF-1, have been found in the promoter region of the visfatin gene. Proinflammatory cytokines including TNF-α and IL-1—known to activate these transcription factors through NF-κB, JNK, and p38—increase the expression of visfatin [75], suggesting the presence of a positive feedback loop for the production of visfatin and regulation of inflammatory responses and stemness by visfatin in cancer cells. The role of visfatin in the regulation of p38 and NF-κB-mediated inflammation and stemness in cancer cells therefore warrant future investigation. Furthermore, limitations of this study include the use of immunodeficient mice, and therefore the complex interplay of visfatin in the context of the immunocompetent host remains to be explored. Although we have examined the role of visfatin in monocyte polarization to M2-like TAMs, further work is required to reveal whether visfatin exhibits a direct role in regulating TAMs to suppress immunosurveillance in the tumor-immune microenvironment.

## 4. Materials and Methods

### 4.1. Cells and Maintenance

Human monocytic cell line THP-1, human breast carcinoma cell lines MDA-MB-231, MDA-MB-468, Hs578T, MCF-7, T47D, and ZR75-1 were purchased from ATCC and Bioresource Collection and Research Center (BCRC, Hsinchu, Taiwan). Peripheral blood mononuclear cells (PBMCs) were isolated using Histopaque-1077 (10771, Sigma-Aldrich, Castle Hill, Australia), from healthy donors who had agreed and signed an informed consent form approved by the Institutional Review Board of Kaohsiung Medical University Hospital (Kaohsiung, Taiwan). MDA-MB-231, luciferase-expressing MDA-MB-231 (Luc- MDA-MB-231), MDA-MB-468, Hs578T, and MCF-7 cells were cultured in DMEM medium (11875093, Thermo Fisher Scientific, Waltham, MA, USA) supplemented with 10% FBS (Biological Industries, Cromwell, CT, USA) and 1% Penicillin-Streptomycin-Amphotericin B Solution (03-033-1B, Biological Industries). THP-1, PBMCs, T47D, and ZR75-1 were cultured in RPMI 1640 medium (12100046, Thermo Fisher Scientific) supplemented with 10% FBS and 1% Penicillin-Streptomycin-Amphotericin B Solution. All cells were grown in a 37 °C incubator under 5% CO_2_ atmosphere at constant humidity.

### 4.2. Flow Cytometry

For characterizing the THP-1 differentiation stage, cells were collected after pretreatment and incubated. Cells were washed with PBS, and resuspended with sorting buffer and analyzed by FC500 Flow Cytometer (Beckman Coulter, Brea, CA, USA).

### 4.3. Indirect Co-culture

After treating THP-1 cells with or without visfatin (0, 100, 200, or 300 ng/mL) for six days, the treated THP-1 cells were indirectly co-cultured with breast cancer cells in a six-well trans-well plate (0.4 μm pores, Corning, Corning, NY, USA). In short, the treated THP-1 suspended in RPMI medium (1 × 10^6^ cells/mL) were seeded in the insert (1.5 mL/insert), and breast cancer cells suspended in DMEM medium (5 × 10^5^ cells/mL) were seeded in the lower well (2.6 mL/well). After 24 h incubation, MDA-MB-231 or MCF-7 cells were collected for evaluating cell viability, migration, invasion, tumorsphere formation, and EMT-related marker expression.

### 4.4. Giemsa Stain and Immunofluorescent Stain

THP-1 and PBMCs were seeded on coverslips and treated with or without visfatin (0, 100, 200, or 300 ng/mL) for six days. For Giemsa stain, THP-1 cells on a coverslip were fixed with methanol and then stained with diluted Giemsa stain solution (GS500, Sigma-Aldrich, St Louis, MO, USA). After washing, the cells were air-dried, mounted with glycerol mounting medium (C0563, Agilent DAKO, Santa Clara, CA, USA) and observed under microscopy. For immunofluorescent stain, THP-1 and PBMCs on coverslips were fixed with 4% formaldehyde and then permeabilized with 0.25% Triton X-100. After blocking with PBS containing 10% FBS, the cells were incubated overnight at 4 °C with rabbit antihuman CD163 antibody (ab87099, Abcam, Cambridge, MA, USA), rabbit CD206 antibody (ab64693, Abcam), or CD11b antibody (ab133357, Abcam) diluted 1:100 in antibody diluent. Secondary hybridization was done with goat antirabbit antibody conjugated with Alex Flour-488 (ab150077, Abcam). Cell nuclei were stained with Hoechst 33258. The CD163^+^, CD206^+^ or CD11b^+^ cells showing green color were observed by fluorescent microscope system (Olympus, Tokyo, Japan).

### 4.5. Cell Viability Assay

After indirect co-culture with THP-1, breast cancer cells were isolated and seeded in 96-well plates (2 × 10^3^ cells/well) and cultured for 24–72 h. XTT (2,3-Bis- (2-methoxy-4-nitro-5-sulfophenyl)-2H-tetrazolium-5-carboxamide, X4251, Sigma-Aldrich, St Louis, MO, USA) assay with PMS (phenazine methosulfate, P9625, Sigma-Aldrich, St Louis, MO, USA) was subsequently performed. 475 nm absorbance (A1) and nonspecific reading 660 nm absorbance (A2) were measured, and the proliferation rate (ΔA) was calculated as A1-A2.

### 4.6. Cell Migration and Invasion Assays

The breast cancer cells were isolated after pretreatment and resuspended in serum-free DMEM. The cells were seeded onto 24-well trans-well inserts coated with or without Matrigel (8 μm pores, Corning). The lower wells were filled with DMEM containing 10% FBS. After incubation for 24 h, cells in the upper chamber were discarded by cotton swabs. The cells trapped in the membrane were fixed and stained with crystal violet and images were acquired by light microscopy. Trapped cells were defined by ImageJ software.

### 4.7. Tumorsphere Formation Assay

The breast cancer cells were isolated following pretreatment and resuspended in phenol red-free DMEM (21063029, Thermo Fisher Scientific). Additional recombinant human fibroblast growth factor basic (20 ng/mL) (100-18C, Peprotech, Rocky Hill, NJ, USA), recombinant human epidermal growth factor (20 ng/mL) (GMP100-15, Peprotech), 1× B27 (17504044, Thermo Fisher Scientific) and insulin (10 μg/mL) were added. The cells were then plated onto an ultra-low-attachment 96-well plate (3474, Corning) (2 × 10^3^ cells/well) and cultured for seven days. >50 μm diameter tumorspheres were counted as positive tumorsphere formation.

### 4.8. Western Blot

Cells were harvested and lysed with RIPA lysis buffer with Protease Inhibitor Cocktail (P8340, Merck). Protein concentration was acquired by using Bio-Rad protein assay (BIO-RAD, Hercules, CA, USA). Proteins were equally loaded into sodium dodecyl sulfate-polyacrylamide gel prior to electrophoresis. After separation, proteins were transferred to nitrocellulose membrane (IBFP0785C, Merck Millipore, Burlington, MA, USA). The membranes were blocked with 5% nonfat milk for one hour at room temperature, and incubated with primary antibodies diluted with 5% BSA at 4 °C overnight. After membrane washing, secondary antibodies were added at room temperature for one hour. The Western blot image was acquired by using chemiluminescence reagent (WBKLS0500, Merck Millipore), and then quantified by ChemiDoc XRS+ imaging system (BIO-RAD). The antibodies we used were listed below: β-actin (GTX109639, GeneTex, Hsinchu, Taiwan), α-tubulin (GTX112141, GeneTex), ZEB1 (GTX105278, GeneTex), OCT4 (GTX101497, GeneTex), β-catenin (ab16051, Abcam, Cambridge, MA, USA), phospho-AKT (4060, Cell Signaling, Danvers, MA, USA), AKT (4691, Cell Signaling), phospho-CREB (GTX61045, GeneTex), CREB (GTX112846, GeneTex), visfatin (ab58640, Abcam).

### 4.9. Cytokine-Chemokine Array and Phospho-Kinase Array

To explore the expression profile of cytokine-chemokine and phospho-kinase in the CM of THP-1 treated with or without visfatin (300 ng/mL), the Proteome profiler^™^ Human XL Cytokine Array Kit (ARY022, R&D Systems, Minneapolis, MN, USA) and Human Phospho-Kinase Array Kit (ARY003B, R&D Systems) were used. The array image was acquired by using chemiluminescence reagent (WBKLS0500, Merck Millipore), and then quantified by ChemiDoc XRS+ imaging system (BIO-RAD).

### 4.10. Elisa

The expression levels of visfatin, CXCL1, PAI-1 and CXCL10 in the CM were detected by using Human PBEF/Visfatin DuoSet ELISA (DY4335-05, R&D Systems), Human CXCL1/GRO alpha Quantikine ELISA Kit (DGR00B, R&D Systems), Human Serpin E1/PAI-1 Quantikine ELISA Kit (DSE100, R&D Systems), and Human CXCL10/IP-10 Quantikine ELISA Kit (DIP100, R&D Systems), according to the manufacturers’ instructions.

### 4.11. Animal Study

All animal experiments were conducted following the Institutional Animal Care and Utilization Committee of Kaohsiung Medical University, Kaohsiung, Taiwan (IACUC number: 102008). The number of animals used was well-designed and minimized according to the three ‘R’s. For the orthotopic mouse model, the Luc-MDA-MB-231 cells were isolated after co-culture with or without V-THP-1 cells, and 2 × 10^6^ cells were injected into female NOD/SCID mice (NOD.CB17-Prkdcscid/NcrCrlBltw; aged six to eight weeks; Lasco, Taiwan) in the fourth mammary fat pads. For the metastasis mouse model, the luciferase-expressing MDA-MB-231 cells were pretreated with or without CM from V-PBMCs for three days, then 2 × 10^5^ cells were injected into female nude mice (BALB/cAnN.Cg-Foxn1nu/CrlNarl; aged six to eight weeks; National Laboratory Animal Center, Taiwan) through the tail-vein. After one week, CXCL1 blocking antibody (10 mg/kg) (AF-453-NA, R&D Systems) was injected intraperitoneally twice per week. The bioluminescent signal from Luc-MDA-MB-231 cells was detected by using the IVIS system; after 45–60 days, the mice were sacrificed for H&E staining and IHC analysis.

### 4.12. Immunohistochemistry

Breast cancer specimens for human tissue microarray were collected from breast cancer patients who underwent surgical treatment at Kaohsiung Medical University Hospital (KMUH). This study was conducted according to the Helsinki Declaration and was approved by the institutional Review Board of KMUH (IRB number: KMUHIRB-F(II)-20170067). The tissue/tumor parts harvested from animals were fixed with formalin and embedded with paraffin to form a tissue block. Four μm paraffin section slides were used to perform immunohistochemistry by using a fully automated Bond-Max System (Leica Microsystems, Wetzlar, Germany). All operation steps were performed in accordance with the manufacturer’s instructions (Leica Microsystems). The primary antibodies used for IHC staining included CXCL1 (GTX53966, GeneTex), phospho ERK (#4370s, Cell Signaling), CK7 (GTX109723, GeneTex), and Ki67 (GTX16667, GeneTex). The staining results of human breast cancer tissue microarray were acquired by using TissueFAXS 3.5 (TissueGnostics, Vienna, Austria), and staining intensity within immune cells was scored as 0, negative; 1, weak; 2, moderate; 3, vigorous.

### 4.13. Statistical Analysis

GraphPad Prism5 software (GraphPad Software Incorporation, San Diego, CA, USA) was applied for all statistical analyses, and all data were presented as mean ± SD. Unpaired *t*-test was applied for two groups comparison, and linear regression was applied for two groups correlation analysis. Statistical significance was marked as follows: * *p* ≤ 0.05; ** *p* ≤ 0.01; *** *p* ≤ 0.001.

## 5. Conclusions

The present study provides new insights into the adipocytokine, visfatin, and its ability to indirectly influence breast cancer progression through monocytic cell differentiation to TAMs, and trigger cytokine CXCL1 secretion to increase the malignant behavior of breast cancer. A positive feedback loop may exist, allowing CXCL1 to induce breast cancer cells to express more visfatin. Adipocytokines pretreated co-culture model, as outlined in the present study, represents the potential of future adipocytokine tumor-stromal research. Breast cancer research based purely on tumor cell lineage models alone should be interpreted with caution, as we have shown that this may result in the incomplete elucidation of tumor pathways, and therefore subsequently translate to reduced efficacy of clinical therapies.

## Supplementary Materials

The following are available online at https://www.mdpi.com/2072-6694/12/12/3526/s1, Figure S1: Breast cancer cells induced THP-1 differentiation, Figure S2: Visfatin combined with PMA induced THP-1 differentiation toward the M2 subtype, Figure S3: Visfatin-treated THP-1 enhanced breast cancer cell viability and invasion, Figure S4: ERK phosphorylation induced by low dose of visfatin in the short-term period, Figure S5: Individual IVIS images of the orthotropic model and metastasis model, Figure S6: Original WB for Visfatin in breast cancer cell lines., Figure S7: Original WB for EMT and stemness markers in MDA-MB-231 cells cocultured with visfatin-treated THP-1 cells, Figure S8: Original WB for visfatin expression in MCF-7 and MDA-MB-231 cells after CXCL1 treatment, Figure S9: Original WB for CREB, pCREB, ERK and pERK expression in THP-1 cells at different time points (0, 30, 60, 90, 120 min) after visfatin (300 ng/mL) treatment, Figure S10: Original WB for CREB, pCREB, ERK and pERK expression in THP-1 cells at 6 days after visfatin (300 ng/mL) treatment, Figure S11: Original WB for CXCL1 and pERK in THP-1 cells pretreated with visfatin (300 ng/mL) for six days, and PD98059 (10 μM) was added on day 3.
