# Supplementary material for: Visfatin Enhances Breast Cancer Progression through CXCL1 Induction in Tumor-Associated Macrophages
Source: Cancers (Basel). 2020 Nov 26;12(12):3526. doi: 10.3390/cancers12123526 (PMC7760195; doi:10.3390/cancers12123526)
Supplement: Supplementary file 1 [file cancers-12-03526-s001.pdf]

cancers

Supplementary Materials:

Visfatin Enhances Breast Cancer Progression through CXCL1 Induction in Tumor-Associated Macrophages

Yen-Yun Wang, Huan-Da Chen, Steven Lo, Yuk-Kwan Chen, Yu-Ci Huang, Stephen Chu-Sung Hu, Ya-Ching Hsieh, Amos C. Hung, Ming-Feng Hou and Shyng-Shiou F. Yuan

Supplementary data

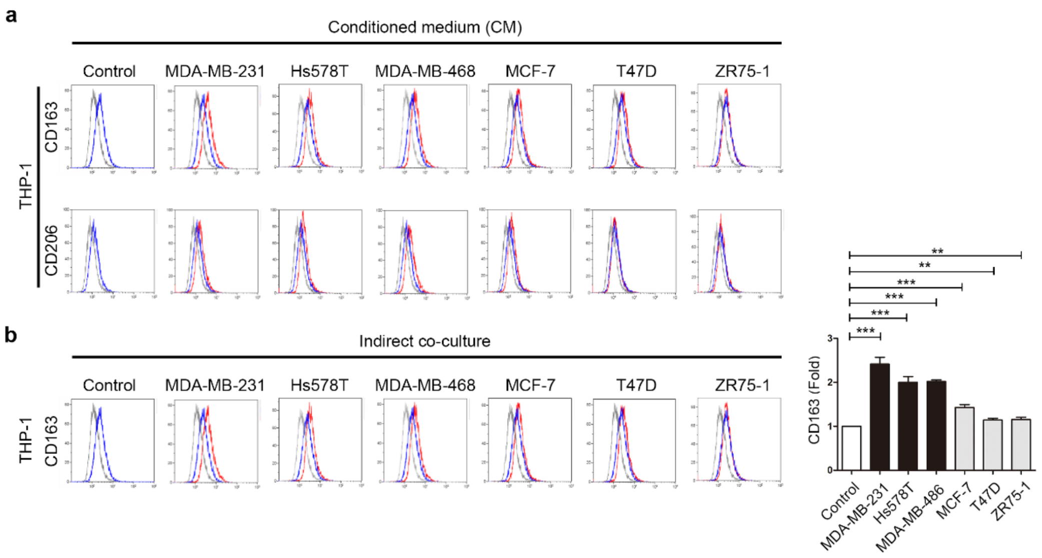

Figure S1. Breast cancer cells induced THP-1 differentiation. (**a**) Flow cytometry histogram plot of Figure S1c. (**b**) Breast cancer cells and THP-1 were indirectly co-cultured for six days (1:1), and the M2 marker CD163 was analyzed by flow cytometry. The t-test was used for statistical analysis. *p*-value ≤ 0.01 marked as **; ≤ 0.001 marked as ***.

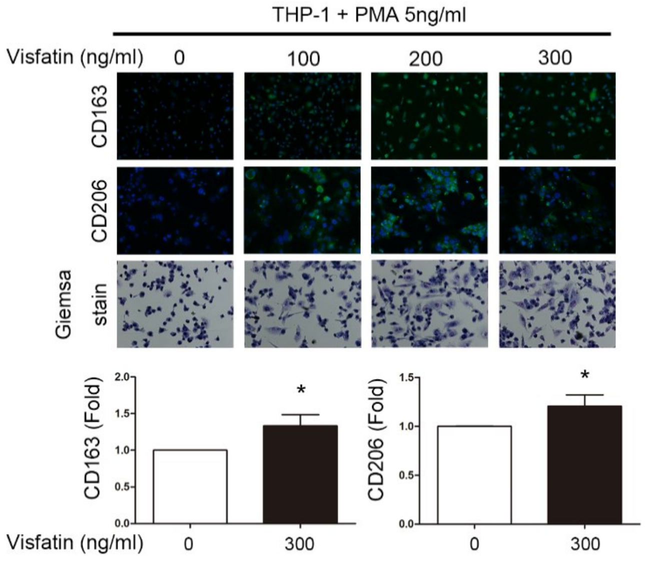

Figure S2. Visfatin combined with PMA induced THP-1 differentiation toward the M2 subtype. PMA is a compound that induces monocyte cells to differentiate into macrophages. THP-1 cells were treated with visfatin at different doses (0, 100, 200, 300 ng/mL) for six days after treatment with PMA for one day, and M2 markers CD163 and CD206 were detected in attached cells by

immunofluorescence staining. Giemsa stain was used to identify macrophages (40×). Statistical analysis was performed by the t-test and the correlation analysis by linear regression. p-value ≤ 0.05 is marked as *.

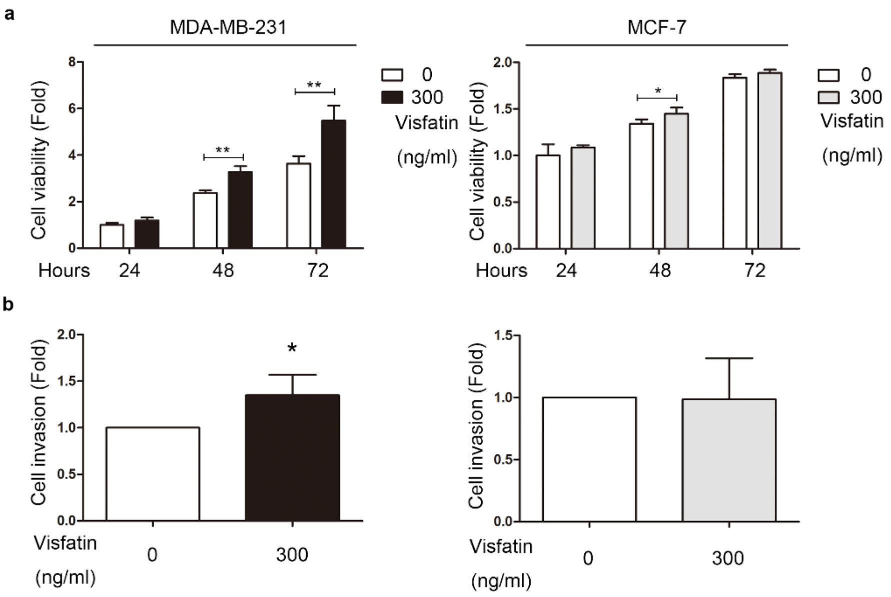

Figure S3. V-THP-1 enhanced breast cancer cell viability and invasion. (**a**) MDA-MB-231 and MCF-7 were co-cultured with V-THP-1 for 24 h. Cell viability was measured for another 24, 48 and 72 h. (**b**) MDA-MB-231 and MCF-7 were co-cultured with V-THP-1 for 24 h, and invasion ability was assessed in a trans-well system. Statistical analysis was performed by the t-test. p-value ≤ 0.05 marked as *; ≦ 0.01 marked as **.

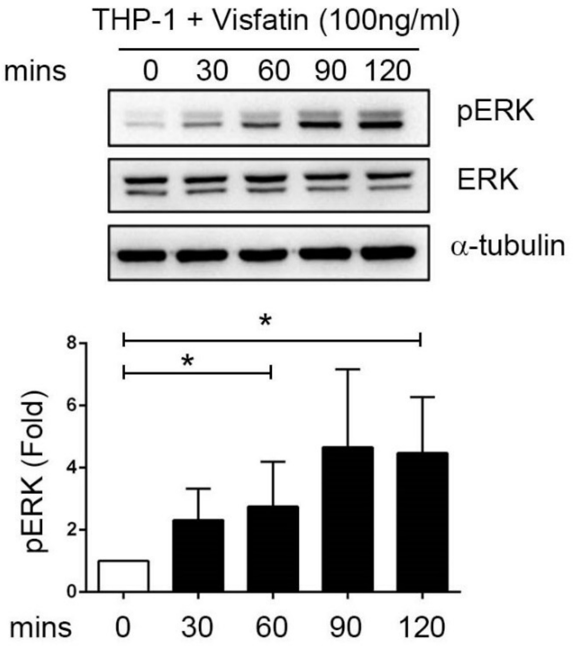

Figure S4. ERK phosphorylation induced by low dose of visfatin in the short-term period. THP-1 cells were treated with visfatin (0, 100 ng/mL) for short-term period (0, 30, 60, 90, 120 min), and phosphorylation of ERK was detected by western blot. Statistical analysis was performed by the t-test. p-value ≤ 0.05 marked as *.

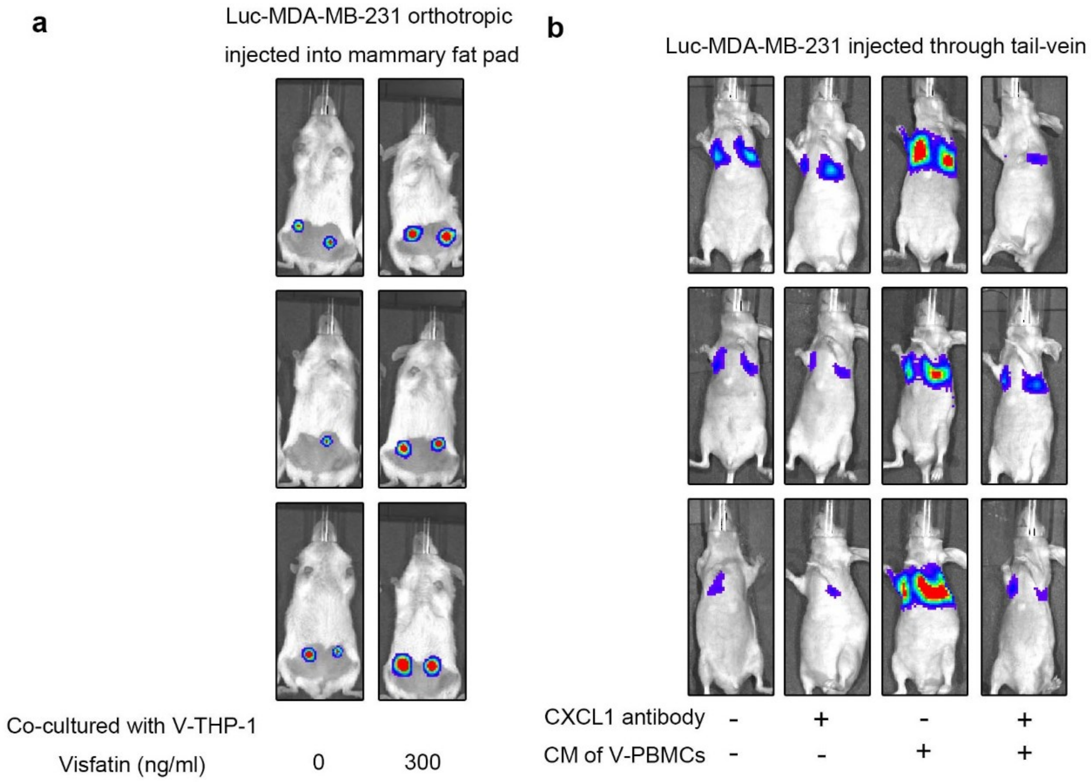

Figure S5. Individual IVIS images of the orthotropic model and metastasis model. (**a**) Three individual IVIS images of a mouse injected with Luc-MDA-MB-231 into mammary fat pad. Luc-MDA-MB-231 was co-cultured with V-THP-1 before injected into the mouse. (**b**) Three individual IVIS images of a mouse injected with Luc-MDA-MB-231 through tail-vein. Luc-MDA-MB-231 was pre-treated with CM of V-PBMCs before injected into the mouse.

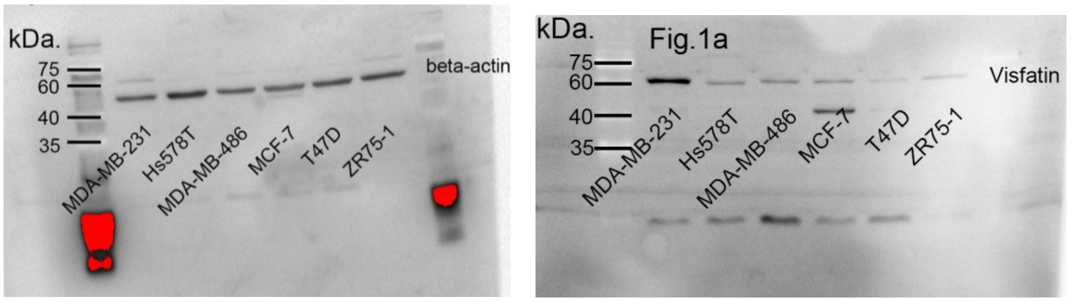

Figure S6. Original WB for Visfatin in breast cancer cell lines.

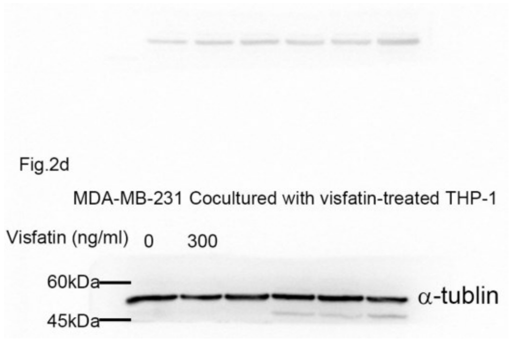

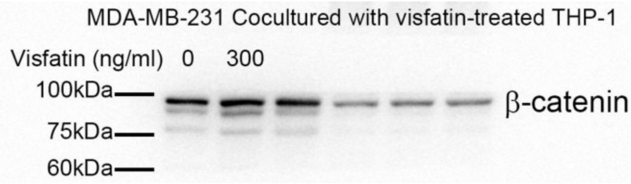

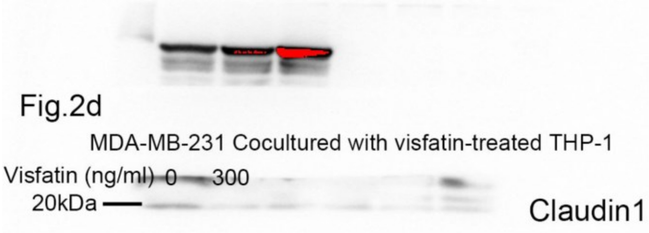

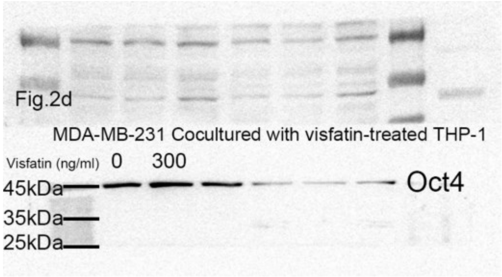

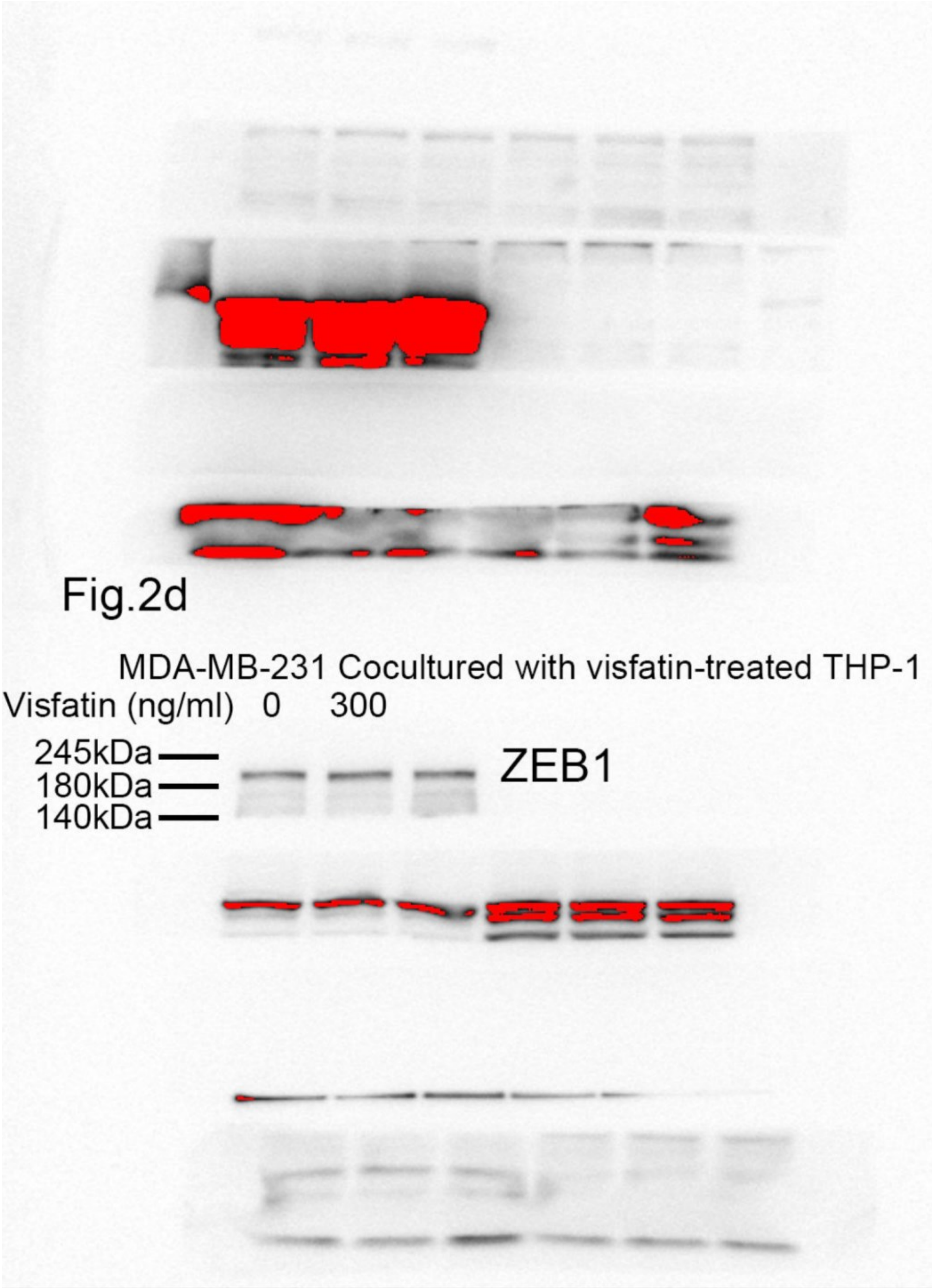

Figure S7. Original WB for EMT and stemness markers in MDA-MB-231 cells cocultured with visfatin-treated THP-1 cells.

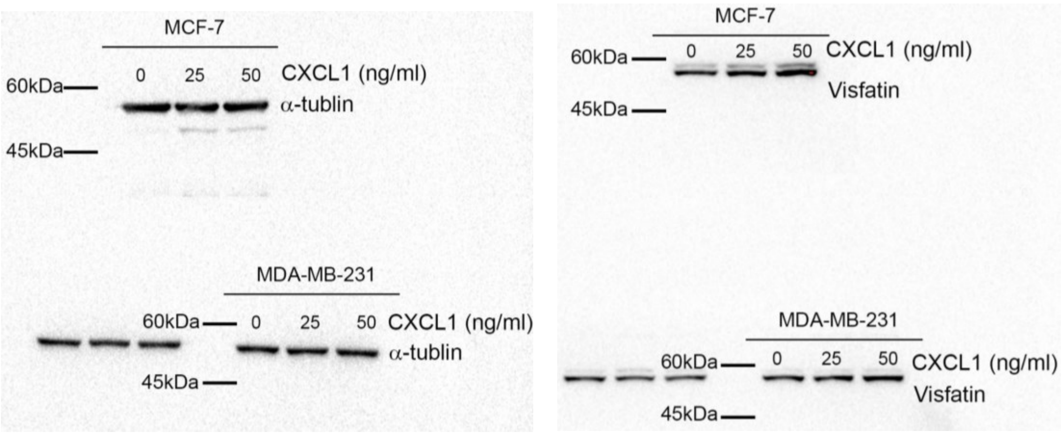

Figure S8. Original WB for visfatin expression in MCF-7 and MDA-MB-231 cells after CXCL1 treatment.

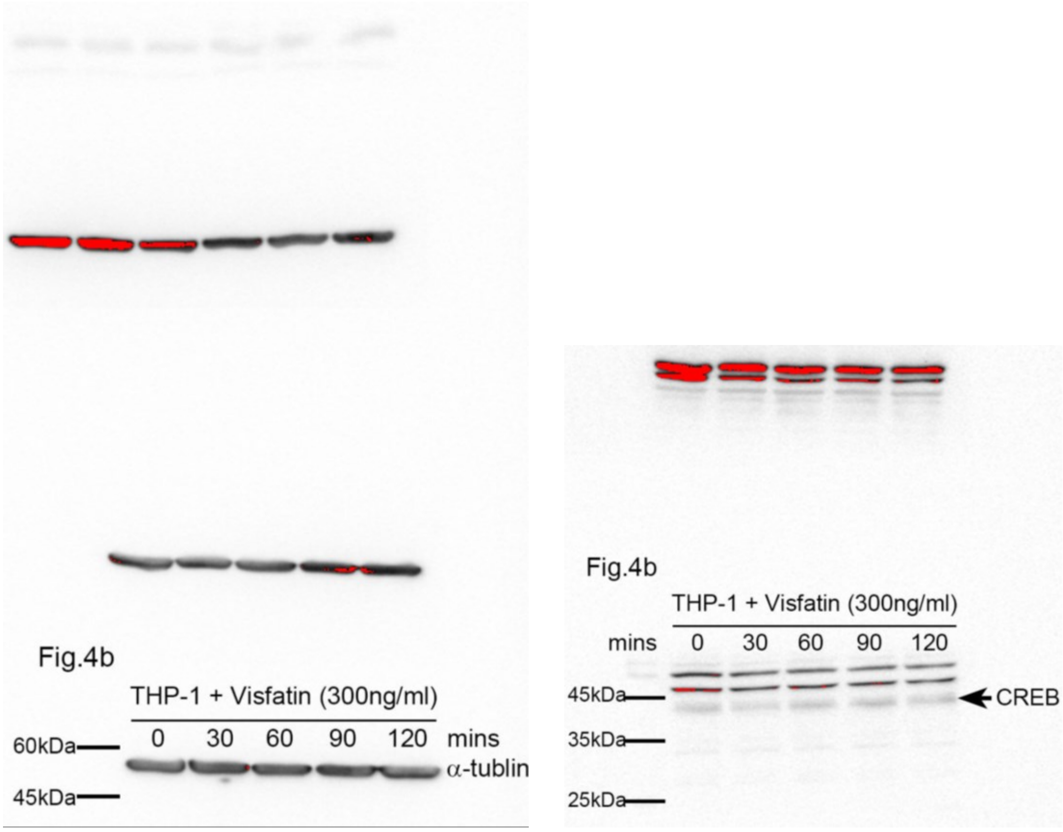

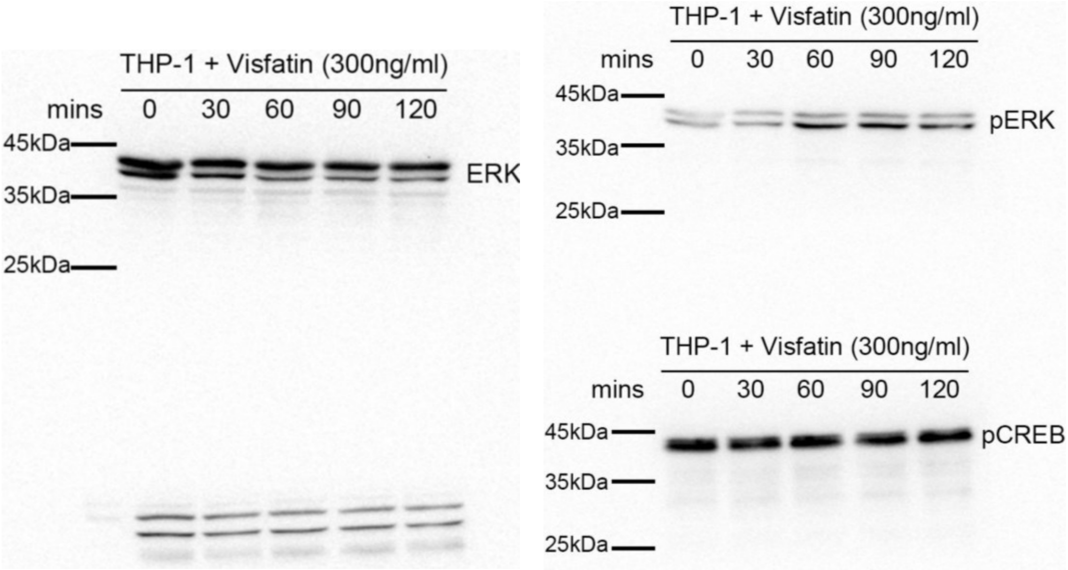

Figure S9. Original WB for CREB, pCREB, ERK and pERK expression in THP-1 cells at different time points (0, 30, 60, 90, 120 min) after visfatin (300 ng/ml) treatment.

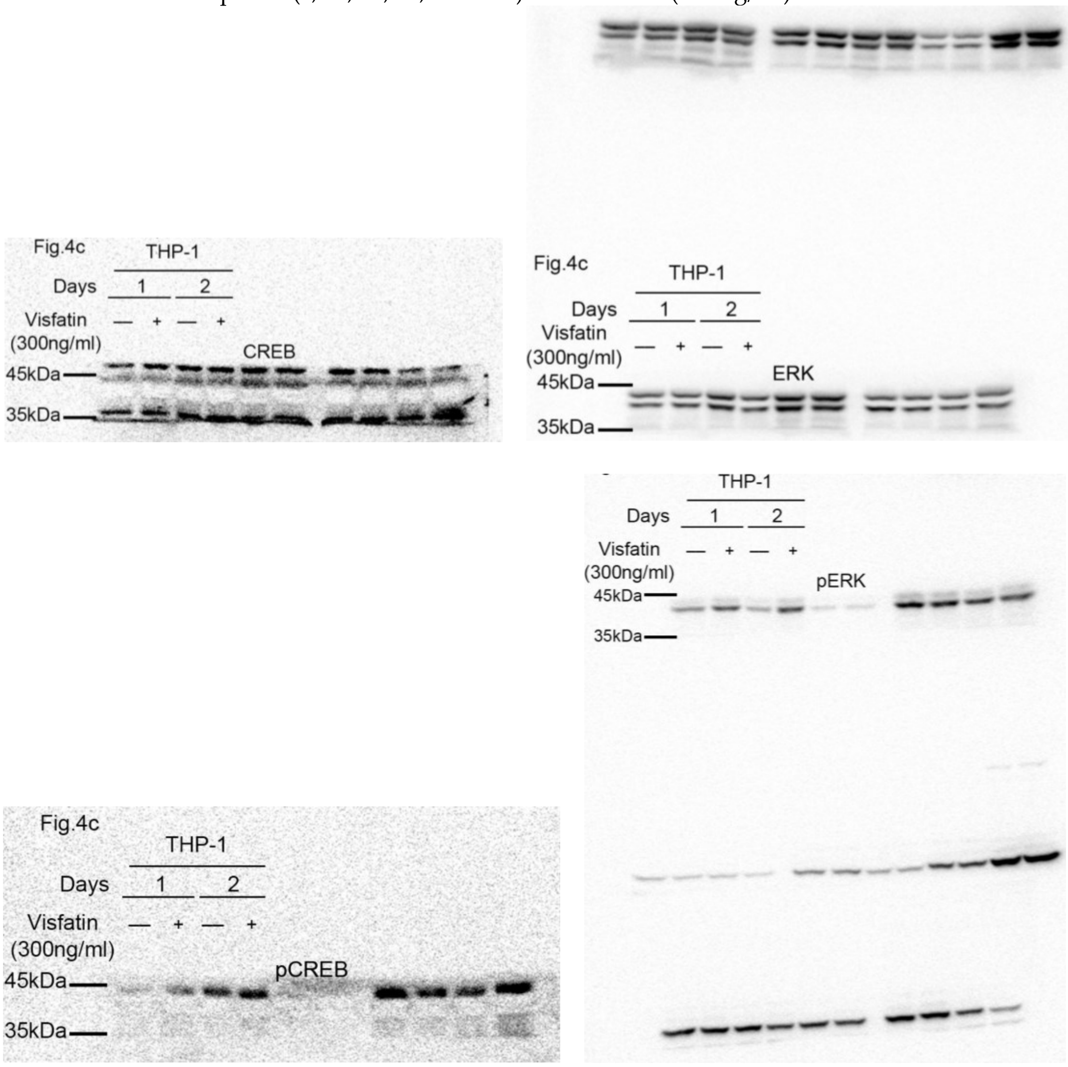

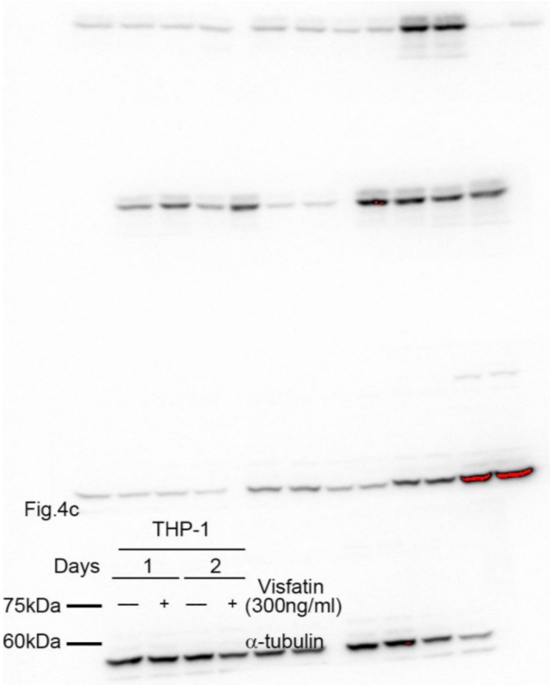
Fig.4c
THP-1
Days
1
2
75kDa
60kDa
— + — +
Visfatin
(300ng/ml)
α-tubulin

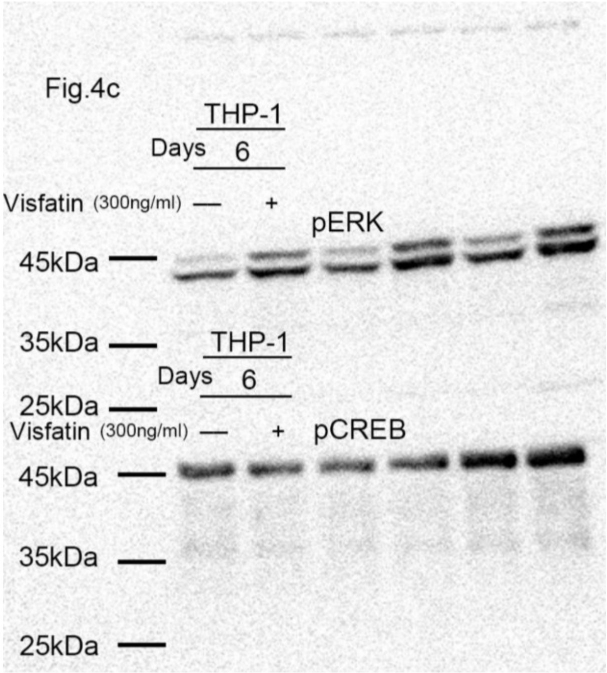
Fig.4c
THP-1
Days 6
Visfatin (300ng/ml) — +
pERK
45kDa
35kDa
25kDa
THP-1
Days 6
Visfatin (300ng/ml) — +
pCREB
45kDa
35kDa
25kDa

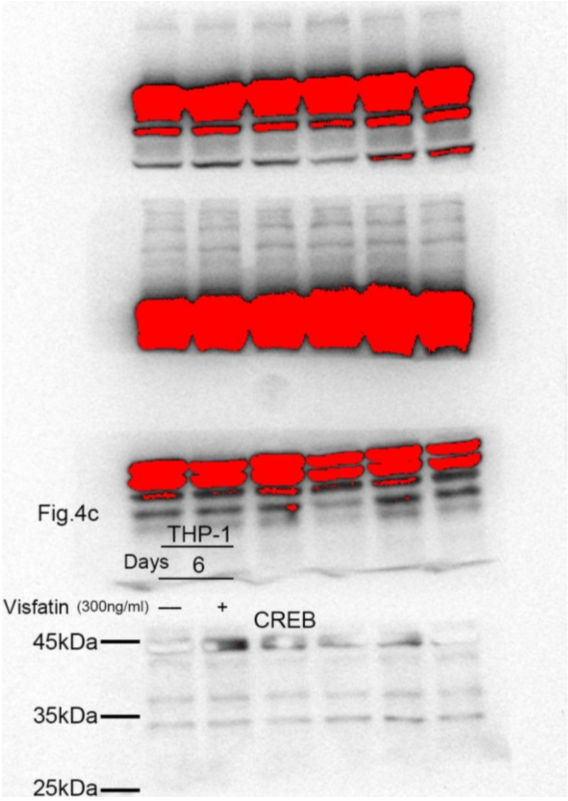
Fig.4c
THP-1
Days 6
Visfatin (300ng/ml) — +
CREB
45kDa
35kDa
25kDa

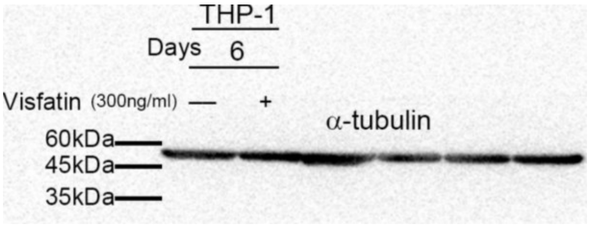
THP-1
Days 6
Visfatin (300ng/ml) — +
α-tubulin
60kDa
45kDa
35kDa

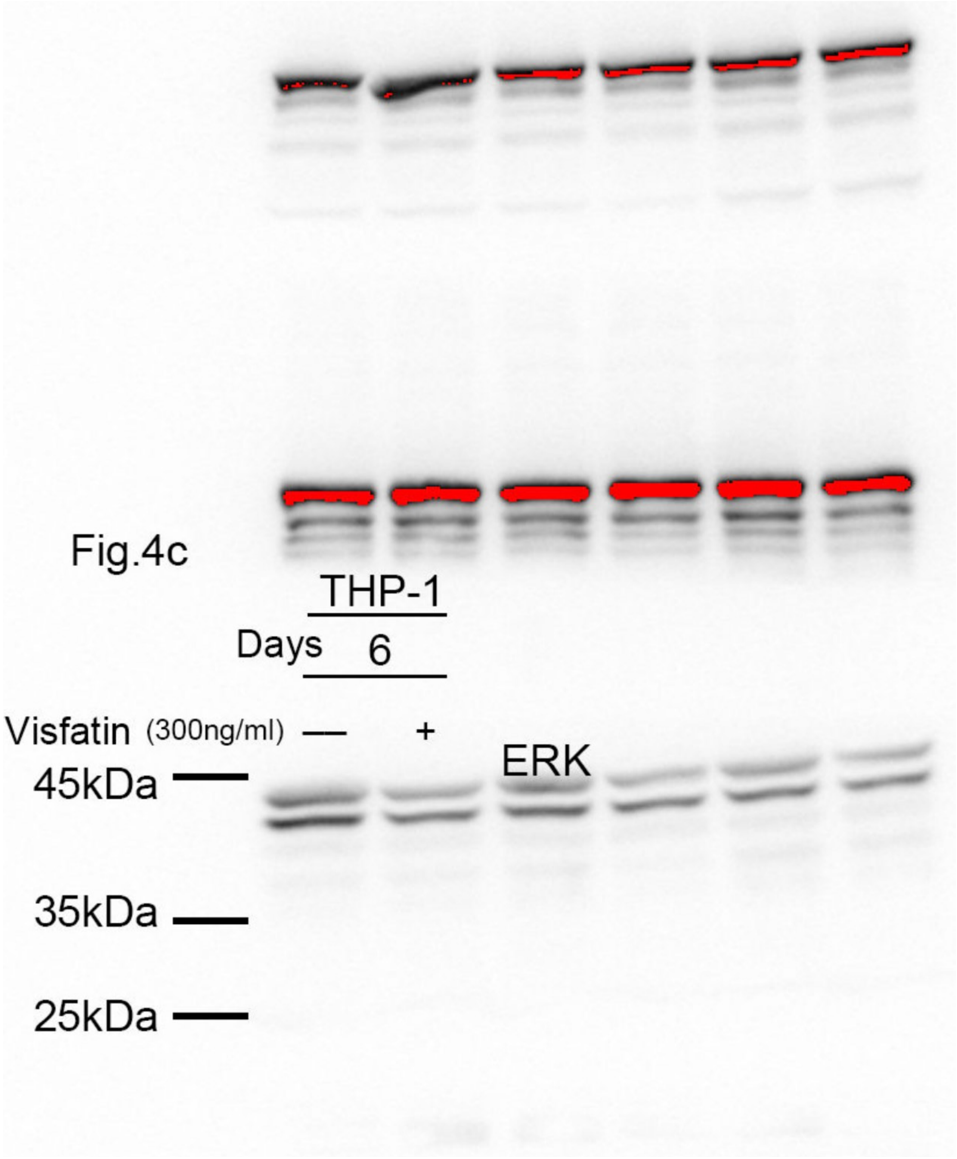

Figure S10. Original WB for CREB, pCREB, ERK and pERK expression in THP-1 cells at 6 days after visfatin (300 ng/ml) treatment.

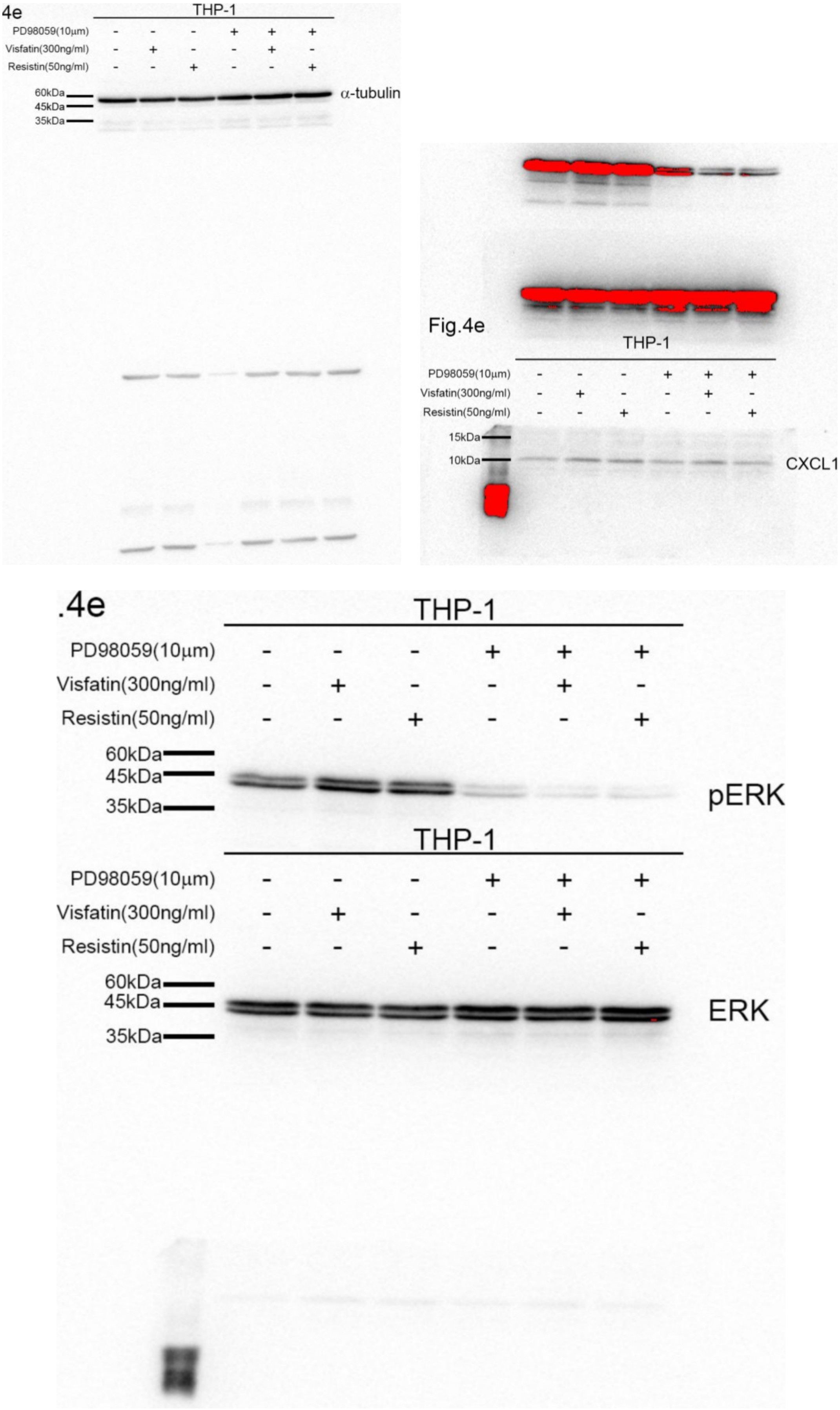

Figure S11. Original WB for CXCL1 and pERK in THP-1 cells pretreated with visfatin (300 ng/mL) for six days, and PD98059 (10 μM) was added on day 3.

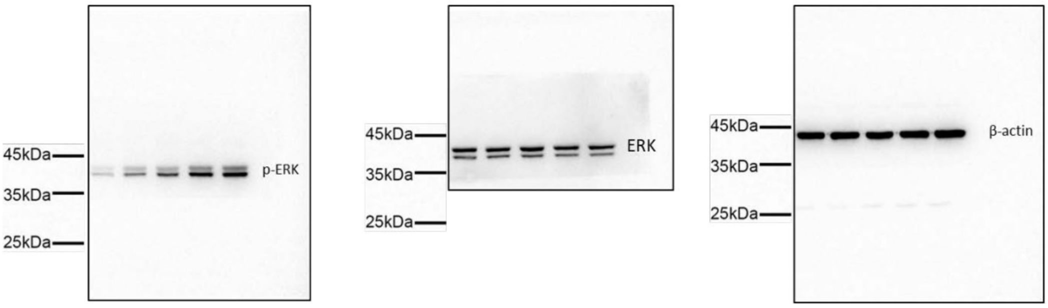

Original WB for figure S4
